# Bone mineral density in midlife long-term users of hormonal contraception in South Africa: relationship with obesity and menopausal status

**DOI:** 10.1186/s40695-018-0035-0

**Published:** 2018-04-10

**Authors:** Mags E. Beksinska, Immo Kleinschmidt, Jenni A. Smit

**Affiliations:** 1 MatCH Research Unit [Maternal, Adolescent and Child Health Research Unit], Department of Obstetrics and Gynaecology, Faculty of Health Sciences, University of the Witwatersrand, 40 Dr AB Xuma Street,11th floor, Suite 1108-9,Commercial City, Durban, 4001 South Africa; 2 London School of Hygiene and Tropical Medicine, Keppel Street, London, WC1E England

**Keywords:** Depot-medroxyprogesterone acetate, Norethisterone enanthate, Combined oral contraceptives, Bone mineral density, Menopause, Follicle stimulating hormone, Body mass index

## Abstract

**Background:**

In South Africa, hormonal contraception is widely used in women over the age of 40 years. One of these methods and the most commonly used is depot-medroxyprogesterone acetate (DMPA) which has been found to have a negative effect on bone mass. Limited information is available on the effect of norethisterone enanthate (NET-EN) on bone mass, and combined oral contraceptives (COCs) have not been found to be associated with loss of bone mass. The aim of this study was to investigate bone mineral density (BMD) in pre and perimenopausal women (40–49 years) in relation to use of DMPA, NET-EN and COCs for at least 12 months preceding recruitment into the study and review associations with body mass index (BMI) and menopausal status.

**Methods:**

One hundred and twenty seven users of DMPA, 102 NET-EN users and 106 COC users were compared to 161 nonuser controls. Menopausal status was assessed, BMI and forearm BMD was measured at the distal radius using dual X-ray absorptiometry. Comparison analysis was conducted at baseline and 2.5 years.

**Results:**

There was no significant difference in BMD between the four contraceptive user groups (*p* = 0.26) with and without adjustment for age at baseline or at 2.5 years (*p* = 0.52). The BMD was found to be significantly associated with BMI (*p* = < 0.0001) with an increase of one unit of BMI translating to an increase of 0.0044 g/cm^2^ in radius BMD*.* Follicle stimulating hormone (FSH) level ≥ 25.8 mIU/mL was associated with a decrease of 0.017 g/cm^2^ in radius BMD relative to women with FSH < 25.8 mIU/mL. Significant interaction between FSH and BMI in their effect on BMD was observed (*p* = .006).

**Conclusion:**

This study found no evidence that long-term use of DMPA, NET-EN and COCs affects forearm BMD in this population at baseline or after 2.5 years of follow-up. This study also reports the complex relationship and significant interaction between FSH and BMI in their effect on BMD. BMD research in older women needs to ensure that women are assessed for menopausal status and BMI.

## Background

In South Africa hormonal contraceptive use is high as reported in the last South African Demographic and Health Survey [1]. Of the two available hormonal injections, older women (> 40 years) almost exclusively use depot medroxyprogesterone acetate (DMPA) (81%) compared to norethisterone enanthate (NET-EN) (19%) [1]. These highly effective methods of contraception may be the method of choice for many women over 40 who have completed childbearing and are concerned about avoiding pregnancy. Hormonal injectables are not generally recommended in perimenopausal women where use of these methods is viewed as “contraceptive overkill” [2]. Most studies have found that current users of DMPA have lower BMD compared to nonusers [3–7]. However, a recent Cochrane review concludes that existing information cannot confirm whether steroidal contraceptives influence future fracture risk [8].

Specifically there is limited information on the effect of hormonal contraception on BMD in women in their midlife (> 40 years of age) [9–12]. As few studies have included women in this age group. Results of these studies have been mixed with some finding no differences in BMD between older DMPA users and nonusers or normal population means [9–11], while one study found a negative effect of DMPA on BMD compared to nonusers [12].

Studies investigating combined oral contraceptive (COC) use in perimenopausal users have not found a negative impact on BMD compared to nonusers of COCs [3]. Two cross-sectional studies have looked at BMD in NET-EN users. In one of these studies [13], current NET-EN users aged 40–44 had lower ultrasound measures in the calcaneus compared to nonusers, while the second study found no difference in forearm BMD between current users and controls [10].

Other potential factors that may play a role in BMD in midlife include menopausal status [14] and obesity [15]. However, evidence suggesting that being overweight / obese may be protective of BMD, is conflicting [16]. This study aimed to investigate BMD in pre and perimenopausal users (40 to 49 years) of DMPA, NET-EN, COC and nonusers of contraception in a 4–5 year follow-up study and review associations with BMI and menopausal status.

## Methods

A cohort of women aged 40 to 49 years old using DMPA, NET-EN, or COCs, and nonusers of hormonal contraception were recruited from a large family planning clinic in Durban, South Africa. For inclusion as a hormonal contraceptive user, women had to have used either DMPA, NET-EN or COCs for at least 1 year. For inclusion in the nonuser control group women should not have used any form of hormonal contraception in the past year. Women who were postmenopausal were excluded from the study at screening using menstrual history (no bleeding for 2 years or more) and follicle-stimulating hormone (FSH) levels from blood samples. An FSH level of ≥25.8 m International Units per milliliter (mIU/mL) was considered to be in the menopausal range (King Edward VIII Hospital Durban; Chemical Pathology Laboratory criteria using Roche Elecsys FSH expected values). Women with an FSH ≥25.8 m (mIU/mL) who reported irregular bleeding within the last 2 years were classified as perimenopausal and were eligible for this study.

On recruitment, a questionnaire was administered to elicit information on lifetime contraceptive history, fertility history, menopausal symptoms and regularity of the menstrual cycle. The examination included height, weight, blood pressure and waist and hip measurement. Forearm BMD was measured by dual energy x-ray absorptionmetry (DXA model DTX-200). Osteometer MediTech A/S Co, Rodovre, Denmark). BMD was measured in grams/centimetre^2^ (g/cm^2^) in the distal forearm (radius). The DXA equipment was standardized daily using a phantom as prescribed by the manufacturers instructions. Accuracy to the standard during the recruitment period was 0.53%. and in vivo precision was 0.94%. Study participants were followed-up at six-monthly intervals for a total of four to 5 years depending on time of recruitment. BMD was measured at each 6-monthly follow-up visit. Final comparison analysis was conducted at 2.5 years as the majority of women (87%) had stopped using a method of contraception by end of study. The study was conducted between 2000 and 2008.

The characteristics of women in the study were quantified as means ± standard deviations (SD), medians, or percentages. FSH levels were divided into two categories according to laboratory cut-off levels for premenopausal (< 25.8 mIU/mL) and perimenopausal/menopausal (≥25.8 mIU/mL). Differences in BMD between contraceptive groups, and the associations between BMD and selected characteristics of the study participants by contraceptive group were assessed using one-way analysis of variance and multiple variable linear regression.

The study aimed to be able to detect a half standard deviation difference in BMD between users and non-users of injectable contraceptives. This would be of biological significance as this difference would translate into a large difference in the risk of fracture in the older woman. Information on the mean and standard deviation of cross sectional measurements of forearm bone mass made in white, European, premenopausal, women as was reported by Nordin [17], was used to estimate sample size. The sample size required for each category assuming a two-tailed statistical test with a probability value (alpha) of 0.05 and with a power (beta) of 0.80 was 63 subjects. Loss to follow up was estimated at 8–10% per year. Sample size was adjusted to 100 per user group to ensure that a statistically significant difference could be detected. Data were analysed using the statistical package STATA (V.12 College station, TX, USA).

Ethical approval was granted by the University of the Witwatersrand, Human Subjects Research Committee (ref M981001), and by the Scientific and Ethical Review Group of the World Health Organization.

## Results

In total, 496 women were recruited. Baseline demographic and reproductive characteristics are summarised in Table 1. The mean age was approximately 43 years in the three hormonal contraceptive user groups with the nonusers on average 2 years older. Almost all women were African except for the COC group which included a higher proportion of Indian and Coloured women. Most women took no regular exercise and the mean BMI of the women in each user group fell into the upper end of obese class 1 (30–34.99 kg/m^2^) group [18]. Women in the contraceptive groups had used their method for approximately four of the previous 5 years prior to recruitment, with all having used the method without a break for the last 12 consecutive months. Only 19.7% of women in the DMPA group and 33.0% of the NET-EN group reported a regular menstrual cycle (between 21 and 35 days), compared with 90.0% of the COC group and 93% of the non-users.

**Table 1:** Baseline characteristics of subjects in the 40–49 year age range by contraceptive method use

Characteristics	DMPA (*n* = 127)	NET-EN (*n* = 102)	COC (*n* = 106)	Non-user Controls (*n* = 161)	*P*-value
Mean age, years (SD)	43.6 (2.7)	43.0 (2.2)	43.7 (2.5)	45.4 (2.5)	< 0.001
Ethnicity %					
African	98.4	95.2	67.0	94.4	0.007
Coloured	1.6	1.0	7.5	2.5	
Indian	0	3.8	25.5	3.1	
Exercise					
No regular exercise (%)	96.9	96.0	94.3	93	0.48
Dieted in last 6 months (%)	0	0	4.7	< 1	0.003
Current smoker (%)	4.7	5.6	5.7	9.9	0.23
Parity (median)	4	3	3	3	0.04
Ever lactated %	88.2	91.3	87.7	83.2	0.34
Mean age at menarche, years(SD)	15.2± 1.7	15.5 ± 1.7	14.8 ± 1.7	14.8 ± 1.6	0.014
Lactation (yrs) median	3.5	3.2	3.0	3.2	0.06
FSH					
< 25.8 mIU/mL, %	72	94	90	68	< 0.0001
≥ 25.8 mIU/mL, %	28	6	10	32	
Use of group method*					
Median use last 5 yrs. (months)	53	45	52	NA	
Median lifetime use (months)	84	49	89	NA	
Median age at first use	36	37	36	NA	
Radius BMD g/cm^2^	0.514	0.514	0.500	0.518	0.26

^*^Only group method shown i.e. group to which women using that method at the time of recruitment were allocated

In total, 48.5% of women in the non-user group were classified as perimenopausal (reported at least one vasomotor symptom in the last 3 months and had an FSH in the perimenopausal/menopausal range). This was a higher proportion than in the other 3 groups (40.2% DMPA, 31.4% NET-EN, 30.1% COCs); however, after adjusting for age, there was no evidence of a difference between the 4 groups in terms of menopausal status (DMPA *p* = 0.48; NET-EN *p* = 0.93; COC *p* = 0.29).

There was no significant difference in BMD at baseline between the four contraceptive user groups at the radius (*p* = .26), with and without adjustment for age (Table 2). Although a small decrease in BMD was noted per year over the age range − 0.0017 g/cm^2^ (95% CI -0.0041-0.0008) this was not statistically significant (*p* = .18). Length of use of method in the last 5 years and total lifetime use was not associated with difference in BMD.

**Table 2:** Factors potentially associated with Radius BMD (unadjusted) at baseline

Factors	Radius BMD g/cm^2^ 95% CI	*P* value
Contraceptive group		0.26
DMPA	0.514 (0.501–0.527)	
NET-EN	0.514 (0.499–0.528)	
COC	0.500 (0.486–0.514)	
Nonuser	0.518 (0.506–0.529)	
Ethinicity		
African	0.516 (0.509–0.523)	
Indian	0.491 (0.451–0.531)	0.007
Coloured	0.479 (0.456–0.503)	
Age, per year	−0.0017 (−0.0041–0.0008)	0.188
BMI, change per kg/m^2^	0.0044 (0.0036–0.0052)	< 0.001
FSH		0.029
< 25.8 mIU/mL, %	0.516 (0.508)-0.524)	
≥ 25.8 mIU/mL, %	0.498 (0.483–0.512)	

Body mass index, FSH level (equal to and above 25.8 mIU/mL versus below 25.8 mIU/mL) and interaction of BMD with FSH level were all significantly associated with BMD in a multiple regression model (r^2^ = 0.2). According to this model, for women of median BMI of 33.9 units, mean radius BMD varied from 0.514 g/cm^2^ [95% CI 0.507–0.521] with FSH < 25.8 mIU/mL, to 0.501 g/cm^2^ [95% CI 0.488–0.513] for women with FSH ≥ 25.8 mIU/mL, (*p* = 0.066). The effect of FSH on BMD was significantly modified by BMI, and vice-versa (*p* = .006). For women with FSH < 25.8 mIU/mL, BMD increased by 0.0038 [95%CI 0.0028–0.0047] (g/cm^2^) per unit increase in BMI, whereas for women with FSH ≥ 25.8 mIU/mL, BMD increased by 0.0067 g/cm^2^ [95% CI 0.0048–0.0086] for each unit increase in BMI (Table 3).

**Table 3:** Results of multiple regression model with BMI and FSH level

Factors	Radius BMD units 95% CI	*P* value
Effect of ^a^FSH (for women of median BMI = 33.9 kg/m^2^)		
< 25.8 mIU/mL	0.514 (0.507–0.521)	< 0.066
≥ 25.8 mIU/mL	0.501 (0.488–0.513)	
Effect of BMI, unit BMD per unit change in BMI For		< 0.001
FSH < 25.8 mIU/mL For	0.0038 (0.0028–0.0047)	
FSH ≥ 25.8 mIU/mL	0.0067 (0.0048–0.0086)	

^a^Significant interaction between FSH level and BMI (*p* = 0.006)

Follicle-stimulating hormone level was found to be significantly different between user groups (*p* = < 0.0001). However, after adjusting for age, the difference between the 4 groups was no longer significant (*p* = 0.13). An increase of 1 year in age increased FSH level by 3 mIU/mL (*p* < 0.001).

Although differences were noted between the contraceptive groups, most were not associated with BMD except for BMI, ethnic group and FSH level. A statistically significant difference in BMD was found between the Indian and African women (*p* = .014); however after adjusting for BMI the difference in BMD was no longer significant.

During follow-up all nonusers of hormonal contraception remained as nonusers, however many women in the user groups continued participation in the study but ceased using a contraceptive method. At baseline 32% of women recruited were nonusers of contraception, this increased to 71% after 3 years of follow-up and by end of the follow-up period the majority of women (87%) had ceased using a method of contraception. Due to small numbers of hormonal contraceptive users from 3 years of follow-up, comparison of user groups was conducted at the 2.5 year visit. At this follow up visit 278 women continued with the same method they were using at baseline. Women were excluded from the analysis if they stopped or changed their method. No difference was found in BMD between the groups at the 2.5 year visit (Table 4).

**Table 4:** Mean Radius BMD at 2.5 years by contraceptive group^a^

	N	Radius BMD g/cm^2^ (SD)	*P* value
Contraceptive group			0.522
DMPA	63	0.511 (.071)	
NET-EN	38	0.501 (.081)	
COC	48	0.500 (.082)	
Nonuser	129	0.504 (.075)	

^a^Only women continuing with the same method from baseline were included in this analysis

## Discussion

This longitudinal study found no difference in forearm BMD between pre and perimenopausal users and nonusers of hormonal contraception at baseline or after 2.5 years of follow-up. This is in agreement with other studies of COC users in the perimenopause, where no change in BMD occurred [3]. The populations investigated in previous studies looking at the effect of DMPA and NET-EN on BMD have included women using the method in their 40s but there is limited information in this age group specifically. The age ranges of women in some studies have included users up to the age of 52, however the numbers have been small. In one cross-sectional study older DMPA users were disaggregated in the data [11] and no differences were found in BMD in women aged between 40 and 49 and a slightly older group of 50–52 compared to a normal population mean in the lumber spine and femoral neck. Tang et al., conducted a 3-year prospective study of perimenopusal women (mean age 43 years) who had used DMPA for 5 years or more [12]. At baseline significantly lower BMD in the spine, femoral neck, trochanter and ward’s triangle were reported compared to never users. At the three-year follow up, women had a mean age of 46 years with a mean length of DMPA use of 10.1 years. At this follow up it was projected that the loss from baseline would be linear, however only small losses were noted of less than 1% in all sites aside from the trochanter where a small increase was noted [12]. The authors of the Tang study concluded that rate of BMD loss may be faster in the first 5 years of DMPA use with a levelling off thereafter. Our DMPA user sample was of similar age and reported length of DMPA use to the Tang study. It may be that users in our study had reached a steady state and further bone loss had not occurred. A further longitudinal study [9] followed up women who had been long-term users of either DMPA or the IUD until menopause. This study found no difference in BMD between these two groups at each of the three forearm sites at one-year follow-up post-menopause.

The absence of differences between the groups in BMD at baseline and follow up in our study is in agreement with some studies of BMD in hormonal contraceptive users in the midlife [3]. Although most studies adjust for BMI as it is a known to be associated with BMD [15], it may also be important to review the overall weight of the sample population to assess the proportion that may be obese. The majority of women in our study were classified in the upper range of obese class 1 which may have conferred some protective effect on their BMD. In 2013, South Africa had an obesity rate of 42% for women and 13.5% for men, the highest overweight and obesity rate in sub-Saharan Africa [19].

Another possible reason for the lack of difference in BMD between the groups may be related to the sensitivity of the measurement using forearm BMD. Forearm DXA has been shown to give good precision [20] and accuracy [21] with the added advantages of equipment that is portable and considerably less cost to purchase and maintain compared to DXA equipment measuring central sites. In a large European osteoporosis cohort, a logistic regression analysis for identification of group (HRT or control), the prediction was best for whole body (82.6%) and spine (80.9%), followed by total hip (78.5%) and lastly, forearm (74.7%). The authors concluded that for clinical diagnosis axial DXA is recommended [22].

The study recruited from a public sector urban family planning clinic in a South African city. There was no locally available public health facility with DXA equipment to measure central body sites due to the high cost of the equipment, maintenance and staff to undertake scans. The Forearm DXA scanner was purchased through the study funding which was only able to cover the cost of a forearm DXA scanner.

Many women may continue to use hormonal injectables into their late forties and beyond menopause as menopausal symptoms such as amenorrhea may be masked by use of progestogen-only hormonal contraceptives which also cause amenorrhea [2]. DMPA has been shown to relieve vasomotor symptoms in perimenopausal women [23, 24]. and DMPA and NET-EN are known to suppress the midcycle surge of follicle-stimulating hormone (FSH) and luteinising hormone (LH), thereby reducing raised FSH levels, although the tonic release of these gonadotrophins continues at luteal phase levels [25]. Data from South Africa presents some evidence that a raised FSH level, although initially supressed, will return to its raised level within the three monthly DMPA and two-monthly NET-EN cycles of use and could be potentially used to assist as a menopausal indicator without interrupting method use in this group of contraceptive users [26]. Detection of menopause or perimenopause does therefore present some challenges in this group of injectable contraceptive users, whose BMD may be compromised by any risk associated with hormonal contraception use.

## Conclusions

This study adds to evidence on the effect of hormonal contraception on BMD in the midlife. This study also reports the complex relationship and significant interaction between FSH and BMI in their effect on BMD. BMD research in older women needs to ensure that women are assessed for menopausal status and BMI.
